# Defining Digital Competence for Older Preschool Children

**DOI:** 10.11621/pir.2021.0411

**Published:** 2021-07-01

**Authors:** Inna A. Kalabina, Tatyana K. Progackaya

**Affiliations:** Herzen State Pedagogical University of Russia, Saint-Petersburg, Russia

**Keywords:** Digital competence, digital devices, parents, preschool children, online risks

## Abstract

**Background:**

Recent studies show that by the age of 5–7, children have already had experience using technology for several years. Discussions about the impact of digital interaction on children are ongoing. Nevertheless, it is becoming clear that there is a need to develop concepts and tools that will help children in the process of exploring the digital world. One of such concepts can be that of digital competence. It refers to the readiness of an individual to apply digital technologies efficiently and safely in various spheres of life.

**Objective:**

To determine the content of digital competence for modern children of older preschool age.

**Design:**

The study included interviewing children, organizing interaction of a child with a digital device, playing a card game, and having their parents answer survey questions.

**Results:**

Our study found that older preschool children had a general idea of how to use digital technologies and could name those functions which they had observed or used themselves. The main purpose of the children’s interactions with digital devices was entertainment. Most of the preschoolers demonstrated low motivation for learning how to use digital devices, and had not developed ideas about how to use digital devices in everyday life safely and effectively.

**Conclusion:**

The concept of digital competence can be applied to the study of issues related to the interaction of older preschool children with the digital environment. The results we obtained can help educators and parents to develop strategies for the appropriate and child-friendly interaction of preschoolers with the digital environment.

## Introduction

The digital environment was not originally designed for children; yet it plays a significant role in children’s lives (United Nations Committee on the Rights of the Child, 2021). According to recent studies, children under two have already used digital devices (Chaudron, Di Giota, & Gemo, 2018; Common Sense Media, 2013; Dardanou et al., 2020). Some parents introduce their children to mobile technologies and tablets at the age of 12–24 months (Archer, Wood, & De Pasquale, 2021), preschool educational institutions actively integrate multimedia in the process of education (Dore & Dynia, 2020), and more and more children are becoming regular Internet users before starting primary school. Children in Russia start using devices at the average age of 3 years old (it varies from 1.5 to 5 years old) (Chaudron et al., 2018).

Most Russian children were born into, or are being raised in, households having two TV sets, Internet access, a tablet, a smartphone, a laptop, a DVD, a home video game console, and other devices (Korotkova et al., 2018). Forty-two percent of children in Russia between 3 and 6 years old have a personal smartphone or tablet, and more than half of all children between 0 and 12 years of age may use their parents’ digital devices and watch TV. Apart from that, preschool children often use digital devices every day with no supervision by their parents (Veraksa, Bukhalenkova, Chichinina, & Almazova, 2020). The average daily time spent on digital activity by Russian children 5–10 years old is 1 to 3 hours (Soldatova & Vishneva, 2019).

While the world of children is gradually digitalizing, what kind of impact the technologies have on today’s children is still a subject of debate. Some research focuses on identifying the challenges of interaction with the digital world on a child: apprehensions about the effect of screen time (Radesky, Schumacher, & Zuckerman, 2015); the negative impact of media and information products (Karabanova & Molchanov, 2018; Tisseron, 2013); and the potential for intensifying traditional childhoodrisks, such as bullying, cyberaggression, and risks related to content; and fuelingnew forms of child abuse and exploitation (UNICEF, 2017; United Nations Committee on the Rights of the Child, 2021). Other researchers are studying the opportunities opened by digital technologies to young members of the digital community: the educational and developing value of apps (Liu, Tan, Huang, Chen, & Liu, 2021; Papadakis, 2021; Papadakis, Alexandraki, & Zaranis); the impact of computer technologies on perception in the learning process (Hoffman, 2014); the development of preschoolers’ executive functions by computer-based technologies (Veraksa & Bukhalenkova, 2017); the development of the creative potential of a gifted child (Bochkarev, 2019); opportunities for learning and education; and access to information (UNICEF, 2017).

Nevertheless, both positive and negative influences are dependent upon the development of skills for the competent use of digital opportunities. Researchers have already well formulated the formal features of the interaction of preschool children with individual electronic devices or resources. However, the perception of the digital world by this age group remains understudied. What motivates children to use particular gadgets? Do they want to learn anything new about the technology? How do they feel in the digital environment? Do they experience any negative effects and how do they cope with them? The answers to these questions are partially known when it comes to adolescents, but there is a gap in the case of preschoolers. For this reason, the values and motivation of preschoolers require research to enable a consistent and purpose-driven education aimed at developing the most important digital competencies.

### The Concept of Digital Competence

Confident and critical use of Information Society Technology for work, leisure, and communication is defined as digital competence (Recommendation of the European Parliament… 2006). Digital competence is regarded as one of the key competencies of a modern person (Cortesi, Hasse, Lombana-Bermudez, Kim, & Gasser, 2020).

To specify the concept, the European Commission’s Joint Research Center has developed the Digital Competence Framework for Citizens (DigComp) (Carretero, Vuorikari, & Punie, 2017; Ferrari, 2013; Kluzer & Pujol Priego, 2018; Vuorikari, Punie, Carretero, & Van den Brande, 2016).

Education researchers are also working on identifying the frameworks and structure for the new term. One of the models of digital competence was designed in the process of elaborating new tools for assessing the complex system of skills and knowledge in the field of computer technology in high school students (Calvani, Fini, Ranieri & Picci, 2008). The authors of the model propose to consider digital competence in its cognitive, technological, and ethical dimensions.

Russian researchers consider digital competence to be “based on continuous acquiring of competencies (a system of relevant knowledge, skills, motivation, and responsibility), the ability of an individual to confidently, effectively, critically, and safely select and apply info-communication technologies in different spheres of life (work with content, communication, consumption, technosphere)” (Soldatova, Nestik, Rasskazova, & Zotova, 2013, p.17). Some works describe this concept as an integral part of social competence. With this approach, the skills and culture of communication on the Internet and safe navigation in the virtual world come to the fore in defining the essence of digital competence (Denisov, 2018).

The social and emotional aspects of using and understanding digital devices have been identified as the basis for digital competence (Ilomäki, Paavola, Lakkala, & Kantosalo, 2016). A fundamental difference between digital competence and digital literacy (or other related terms) is the inclusion of a motivational component in the essence of the concept, which determines, inter alia, its psychological and pedagogical orientation.

### Model of Digital Competence of Older Preschool Children

To date, researchers have conducted in-depth studies of the problems involved in the development of digital competence in adolescents; however, the digital competence of children of other age groups has not yet been studied. More specifically, even though several models of digital competence already exist (Calvani et al., 2008; Carretero et al., 2017; Ferrari, 2013; Soldatova et al., 2013), no model has yet been developed for preschool children. To study this age group, the model proposed by the researchers of the Foundation for Internet Development is more applicable; specifically, a model which includes the components of knowledge, skills, motivation, safety, and responsibility (Soldatova et al., 2013). In our opinion, it is possible to speak about acquiring the basics of digital competence in children of older preschool age. In this age cohort, motivation and responsibility become the central components of digital competence as a psychological and pedagogical category. Nevertheless, the age characteristics of this group of children require clarification of these components of the model.

In our study we viewed motivation as the pursuit of improving one’s literacy. However, in studying the motivational component in older preschool children, we were not only interested in the wish or unwillingness to learn how to use digital technologies, but also in the children’s specific motives, be they cognitive, game-related, external, social, etc. At the age of 5–7 years, a child cannot bear full responsibility for his/her actions and is unable to ensure their technical safety. For this reason, our study identified the principal component of the digital competence of an older preschool child as the awareness of the family rules for using digital devices, and the behaviors which the child chooses when faced with technical problems and online environment risks.

It must be also clarified that, from our point of view, the level of skills and the ability to independently use familiar digital devices, and learn how to use unfamiliar ones, are not very important in older preschool age, because they are directly associated with the duration and intensity of interaction with digital devices. For this age category, it is more relevant, in our opinion, to address the existing experience of using digital devices in various spheres of a child’s life.

The meaning of the components of digital competence of older preschool children, which we adhere to in this work, can be presented in the form of a table (*Table 1*).

**Table 1:** Digital competence of older preschool children

Component	Content
Knowledge	Knows a variety of uses for digital devices and understands that digital devices can be used for more than just entertainment purposes Has an idea of the existence of various risks in the digital environment, including online risks Has an idea of how the Internet functions and understands that they are an Internet user
Skills	Has the minimum skills required to use digital technologies as a tool for self-studies, self-development, creative activity, and communication
Motivation	Is motivated to learn how to interact with digital devices for pragmatic purposes Has motivation, which includes both external and internal motives
Safety and responsibility	Knows the family rules for the use of digital devices and tries to comply with them When facing risks in the digital environment, asks for help from an adult/parent

## Methods

### Aims and Research Questions

Our research aimed to explore how the content of digital competence can be defined for modern children of older preschool age. The study sought to determine the particularities of perception and use of digital technologies, areas of knowledge about technologies, motivation for the use of technologies, and ideas about safe behavior when using technologies, shown by this age group. The study also examined the attitude of parents toward the use of technology by their children, and their strategies and methods of parental control over it.

The main questions this study sought to answer were:

- How often and for what purposes do older preschool children use technology?
- What knowledge do children have about the world of technology?
- How do children assess themselves as digital users? Do they want to improve their digital competence?
- How do children relate to technologies? What feelings and emotions do they experience when using them?
- How do older preschoolers understand security in the digital world, and how do they deal with online risks?
- How do parents view the use of technology by their children, and for what purpose do they give and buy digital devices for their children?

### Participants

The study involved two groups of respondents: 1) children of older preschool age who attend kindergarten and live in St. Petersburg, and 2) their parents. Participants were selected from families enrolled in different education groups at the same public kindergarten. Simple random sampling was used. The employees of the kindergarten were involved in the dissemination of information about the research to be conducted.

Parents who did not answer all the questions in the questionnaire were excluded from the sample. The final number of parents who participated in the study was 43. Twenty-four (55.8%) of the respondents were parents of girls, and nineteen (44.2%) were parents of boys. Forty-one (95.3%) were mothers, and only two (4.7%) of the respondents were fathers. The age of the adult respondents ranged from 24 to 47 years. Thirty-two (74.4%) of the respondents had higher education.

The answers of the children-participants during the interviews were included regardless of their completeness. The total number of children involved was 41. Ofthese, 61% (25) were girls; 39% (16) were boys. The ages of the respondents were distributed as follows: respondents age 5 years = 41.5% (17); 6 years = 26.2% (11); and 7years = 31.7% (13). Of the total number of respondents, 61% (25) had older siblings, and 39% (16) did not.

### Procedure

Our study was based on the materials used in the international research on the interaction of children from 0 to 8 years old with digital technologies (Chaudron, 2015), and on a questionnaire developed by the Foundation for Internet Development in collaboration with the Levada Center for the all-Russian study “Digital competence of adolescents and parents” (Soldatova et al., 2013). The first was used as a basis for drafting the interviews with the children; the second was used as a basis for compiling the questionnaire for parents. Both survey instruments were adapted for the purpose of this study: the age characteristics of the children’s group were taken into account; questions to respondents were structured according to the components of digital competence.

The researchers interacted with the children in an environment with which they were familiar: in the child’s room at home or in the playroom of the kindergarten they go to. The interview with each respondent took about 25-30 minutes. Before getting to the main part of the interview **(***i.e.,* to identify the children’s preferences**)** and in order to make the child comfortable**,** a card game was used. The cards featured images of various digital devices and electronic or traditional toys. In the course of the game, the respondents were asked to place cards on the scale “Like/use”–”Don’t like/don’t use**,**” which helped us to understand how important digital technologies were in the children’s lives, compared to other elements of modern childhood.

In the main part of the interview, thematic sets of questions were asked in order to identify the selected components of digital competence:

1. A set with questions about the child’s user activity: digital devices, applications, and Internet resources used, the frequency and intensity of use, and purposes of use: “*Which of these devices do you have at home*?”; “*Which of them can you use*?”; “*What can you do with this device*?”; “*Which of these activities is your favorite*?”; “*Which applications in this device can you call your favorites*?,” etc.
2. A set with questions aimed at identifying the child’s experience in using digital devices in various fields of technology application: “*Do you use this device alone, or does someone help you*?”; “*How did you learn to use this device? Who helped you with this?*”; “*Do you know how to study/buy something, take a photo, make a video, record a song, draw a picture, etc./communicate with someone using this device?”; “Can you show me how you usually do it?*,” etc.
3. A set of questions aimed at identifying the respondent’s knowledge about digital devices, including the areas and methods of the application of technologies, and the impact on people and society: “*Can this device be used to study, buy something, take a photo, make a video, record a song, draw a picture, etc./communicate with someone using this device?” “How would you explain what the Internet is? ”; “Why do people use it?,”* etc.
4. A set of questions aimed at identifying the child’s motivation for learning to use digital devices, and his/her specific motives, as well as identifying the child’s attitude toward digital devices: *“Do you think it is important to learn how to use this device?”; “Do you still need to learn or do you already know how to use it?”; “Why is it important to learn to use this device?”; “How would you explain why you use this device?”; “In your opinion, does this device bring more good or bad things to people? And to you personally?*,” etc.
5. A set of questions about safety when using digital devices, including knowledge of family rules, the pursuit of complying with them, and a model of behavior in case of facing online risks: *“Do you use this device alone, or does someone help you?”; “Can you use this device for as long as you want? If not, why not?”; “Can you use this device everywhere?”; “Can you use this device alone or should someone be with you?”; “Are there any rules for using this device? Who sets these rules? Do you follow them? What happens if you break any of them?”; “Have you ever felt unpleasant/scared/sad when you used this device? What made you feel so?”; “What did you do in this situation?,”* etc.

At the end of the interview, in order to identify the child’s skills in using a digital device, the interviewer asked participants to demonstrate their skills when using a tablet (iPad). The process of using the device was monitored.

The questionnaire for parents consisted of 39 questions, including dichotomous choice (yes/no), multiple-choice, open-ended, and closed-ended questions. Collection of the parents’ answers was organized through the online-service Google Forms. Respondents were asked questions about the types of digital devices used by their child; whether their child has a personal digital device; the length of time their child uses digital devices on weekdays and weekends; the purposes of the use of digital devices; the attitude of the parents toward digital technologies; and the methods of mediation used by the parents to regulate their children’s interaction with digital devices.

## Results

### Use of Digital Devices by Children Ages 5–7

The study showed that 95.1% of children had the experience of active interaction with various digital devices. Even if their parents did not allow their children to actively interact with these technologies, the children had a positive attitude towards them, but at the same time they had no idea about the rules for their safe use. Also, 68.3% of the children in the survey had their own digital devices (*Table 2*).

**Table 2:** Types of personal digital devices of preschool children ages 5–7

Digital device	of Number responses	of Percentage responses
Smartphone	15	37
Tablet	14	34
Smart Watches	7	17
Mobile Phone	4	10
Laptop	1	2
Game console	1	2

These older preschool children could not set the duration of the use of digital devices by themselves, which means they could not control it. According to the parents’ answers, 61% of their children used digital technologies mostly between 1 and 3 hours a day on weekdays. On weekends, the duration of use of digital devices by this age group increased significantly (*Table 3*).

**Table 3:** Duration of use of digital devices by preschool children ages 5–7

Duration of use	Weekdays	Weekend
Less than an hour	13 (32%)	7 (17%)
1–3 hours	26 (63%)	25 (61%)
3–5 hours	3 (7%)	8 (20%)
5–8 hours	0	2 (5%)
Difficult to answer	1 (2%)	1 (2%)

The main purpose of using digital technologies by older preschool children was entertainment. The children could also use the technology for educational purposes, but usually not by their own choice, not regularly, and not often (*Table 4*).

**Table 4:** Ways of using digital devices by older preschool children

Way of use	of Number responses	of Percentage responses
Watching movies and cartoons	41	100
Playing video games	34	83
Watching videos	17	42
Taking photos	16	39
Communication	12	29
Drawing	9	22
Making videos	6	15
Listening to music and fairy tales	3	7

When asked about their favorite multimedia content, these older preschool children said they could watch cartoons, movies, and different video content available on platforms such as YouTube, TikTok, and Likee.

### Knowledge, Skills and Motivation as Components of Digital Competence of Preschoolers

The knowledge of the digital world and technology which the majority of these older preschool children possessed can be described as fragmentary and unsystematic. More specifically, the majority of respondents had an idea of only a few areas of life and activity where digital devices could be used and could rarely list them on their own. Only in a few cases had the children developed a concept of what the Internet is, although they were active Internet users (*Table 5*).

**Table 5:** Older preschool children’s ideas of the Internet

Child’s definition	of Number responses	of Percentage responses
Electricity for digital devices	9	22
Something that turns on cartoons or games	9	22
Communication tool	3	7
Something that could be distributed by digital devices	3	7
App	2	5
Information storage and transmission system	2	5

These older preschoolers showed varying levels of confidence in interacting with digital devices. In most cases, the children succeeded in operations which required touch screen use (*Table 6*). Slightly more than half of the children could type a text using a touch keyboard, but only in isolated cases did the children demonstrate proficiency in specific skills, such as drawing or voice typing.

**Table 6:** Results of monitoring the use of touch screen digital devices by older preschool children

Operation with a digital device (iPad)	of Number responses	of Percentage responses
Choose the way to interact with the screen (press, swipe, etc.)	41	100
Use icons to select and get access to applications	39	95
Flip the page and access the content with a swipe from right to left or top to bottom	39	95
Resize content (increase, decrease)	36	88
Choose the icon to activate the application	35	85
Select and move content in the app	34	83
Swipe the screen to get access to additional pages with apps	33	81
Find and press the button to turn on the device	32	78
Swipe the screen to get access to apps and enter passcode if needed	30	73

More than half of the respondents — 53.7% (22) — believed that they had already fully learned how to use these devices; that is, they had low motivation to increase their digital competence. It can be assumed that older preschool children tend to perceive their level of interaction with digital devices as high, due to the fact that they independently use only a limited number of functions, even on their own digital devices. Analysis of the children’s answers allowed us to identify the following groups of motives for increasing digital competence:

- Interest in the adult world. An emphasis on self-image as an adult: *“You need to study so that later, when you become an adult, you already know how to work”; “You need to learn to use. For example, you have an important conference of some kind, but you cannot hold on a push-button telephone or TV. And you have to learn.”*
- Social motives. An emphasis on fear of taking a socially disapproving act, or harming yourself or others: *“You have to study, because if you press the wrong button, the money will be withdrawn there, or something else will happen. Or it will explode”; “When you press all the buttons in a row, it can deteriorate. And never work again. Therefore, you have to learn.”*
- Communicative motives. An emphasis on mastering the communicative function of technology: “*I think it’s important for people to learn this. You must have WhatsApp to be able to call another person.”*
- Play motives. An emphasis on mastering the entertainment function of technology: *“You need to learn. For example, I asked my parents about what to press on the phone and where it was, and what games to download, how to download games”; “I saw in one video about games where they somehow turned on the joystick and played on the tablet. I would like that too.”*

### Safety of Children Ages 5–7 in the Digital World

Older preschool children often find it difficult to cite the family rules for using digital devices. Nevertheless, more than half of the surveyed respondents expressed their readiness to follow the rules set by their parents. Some of the children, however, admitted that they did not even comply with the clearly set rules. Some others reported that they did not agree with the rules and were reluctant to follow them.

More than 46 percent (46.3%) of the respondents gave a positive answer to whether a particular digital device could be dangerous. However, the children’s answers were mainly related to the physical characteristics of these objects: *e.g*., the device can break, or fall into the water and cause an electric shock to a person. The rest of the respondents could also recall some negative experiences of using digital devices and even facing online risks, but they did not speak about these situations as potentially dangerous. At the same time, when faced with various risks of the online environment, the children preferred not to inform their parents about them and tried to cope with the problems on their own. Most often this was due to the children’s fear of being punished.

### The Attitude of Parents Towards the Use of Digital Technologies by Their Children

The parents of these older preschool children perceived digital technologies in their children’s lives primarily as a means of entertainment, relatively speaking, “toys.” At the same time, a significant number of parents were aware of the potential of digital devices for their child’s development, but this potential was rarely realized (*Table 7*).

**Table 7:** Advantages of using digital technologies for older preschool children according to parents

Advantage	Number of responses	Percentage of responses
Information	17	40
Development and learning	17	40
More effective learning technologies	3	7
Communication with parents	3	7
Communication	2	5
Unlimited possibilities	1	2

Moreover, the parents of the preschoolers tended to underestimate the risks of the online environment to their children (*Table 8*).

**Table 8:** The risks already faced by their children ages 5–7 in the online environment according to the parents

Online-risk	of Number responses	of Percentage responses
Confident that children did not face risks	24	56
Found it difficult to answer	8	19
Malware	5	12
Content containing scenes of violence, cruelty, murder	5	12
Insult, humiliation, harassment, or resentment	4	9
Promotion of alcohol, drugs, or tobacco	2	5
Become a victim of fraud or money theft	2	5
Theft of personal data	1	2

A significant number of parents also found it difficult to give specific answers to questions about ensuring their children’s safe interaction with digital devices (*Table 9*).

**Table 9:** How parents regulate their child’s interaction with various digital devices

Parental mediation method	of Number responses	of Percentage responses
Limiting the time of use	31	72
Supervision while using a digital device	27	63
Talking about digital devices	22	51
Monitoring the use of digital devices	22	51
Establishing rules for the use of digital devices	21	49
Co-using of digital devices with child	20	47
Teaching the child to use digital devices	17	40
Banning the use of a digital device	16	37
Telling the child about the use of digital devices and the Internet	13	30
Using special software for monitoring	10	23

## Discussion

The data we obtained allows us to determine several features in the digital technology use by older preschool children. A significant percentage of children ages 5–7 periodically interacts with various digital devices. The most popular devices were the smartphone and tablet. These results are similar to those of other studies (Dardanaou et al., 2020; Papadakis et al., 2021). The duration of digital device use by the children exceeded the time recommended for this age group and generally ranged from 1 to 3 hours a day, a result which is consistent with data reported in other studies (Soldatova & Vishneva, 2019). Children could use their parents’ digital devices, family digital devices at home, and their own digital devices. More than half of the children had personal gadgets. This number is higher in comparison with the studies previously conducted in Russia. It is also noteworthy that despite the presence of digital devices in kindergarten, during the study the children did not mention the experience of interacting with them.

Older preschoolers primarily used digital technologies to watch movies and cartoons and to play video games. Also, quite often children watched videos on video hosting and short videos on social media. Entertainment as the most preferred use of digital devices and apps by children has also been described in a number of other studies (Chaudron et al., 2018; Veraksa et al., 2020).

At the same time, the preferred game content differed according to the gender of the respondents. Boys were more likely to choose multi-level arcades, strategy, and online games with characters, where points must be collected, and opponents must be defeated. Girls were more likely to choose games without a plot where they had to take care of a computer character, or creative games such as drawing games, coloring books, character constructors, etc. Boys more often interacted with in-game content inappropriate for their age group (Brawl Stars, Among Us, Fortnite, War Thunder, etc.). The games they named often contain elements of cartoon violence and involve acquisition of additional capabilities by characters for real money. One game which was mentioned by the respondents of both genders was Minecraft. This correlates with data from other studies, which explored differences in preferred activities with digital devices among boys and girls, as well as the content chosen (Brito & Dias, 2019; Veraksa et al., 2020). Girls were more communication-oriented: they were more likely to cite “connecting, communicating” as their favorite digital device activity, and chose drawing, music, educational activities, and watching cartoons with a digital device more often than boys (Veraksa et al., 2020).

When it comes to the specifics of digital device perception by older preschool children, it should be noted that their parents viewed digital technologies in their children’s lives primarily as a means of entertainment. Parents’ answers indicated that they rarely used digital technologies to stimulate their child’s learning and development, although they knew about such potential. This led to the children developing the same attitude. While the children were predominantly positive about the technology, they were rarely able to explain how digital devices are beneficial to them personally and to the society as a whole. Also, it can be assumed that their attitude was shaped by the mostly positive emotions (joy, fun, and interest) which they had experienced, since they used the gadgets to play and to watch entertainment.

The knowledge these older preschool children had about the digital world was limited mostly to the general idea of the spheres of application of digital devices, and the functions which the children had observed or used themselves. Respondents had the basic skills required to interact with a touch screen. Competence in tablet use by young children has also been shown in studies of various European nations (Chaudron et al., 2018). Also, according to our data, the older preschool children generally demonstrated low motivation for learning how to use digital devices, since they had a limited understanding of the functions of technologies which were available. The responses of motivated children allow us to suggest that when children are better informed about their opportunities in the digital world, their motivation could increase. Children of this age group were also virtually unaware of the possible dangers of the online environment, which gave them the false impression that they could cope with any problem if it arose. Although these older preschool children used various digital devices actively, most of them did not have any clear idea about how to use them safely and effectively in their everyday lives.

Moreover, the majority of the children in the study chose not to tell their parents about their negative experiences while interacting with digital devices, since they were afraid of subsequent punishment. The same problem has been shown in the UNICEF report. While attitudes vary by culture, children often turn first to their peers when they experience risks and harm online, making it harder for parents to protect their children (UNICEF, 2017). Moreover, the parents in our research tended to underestimate the risks of the online environment to their children. Most parental moderation strategies involved limiting the time of use of digital devices. Other studies also confirm this finding (Papadakis et al., 2021).

Parental supervision while using a digital device was also frequently mentioned by parents, but it is hardly possible all the time, considering the duration of their children’s use of digital devices. Co-usage of digital devices and lessons on how to use devices were effective but uncommon practices among parents. According to other Russian studies, preschool children often use digital devices every day with no supervision by their parents (Veraksa et al., 2020). Older preschoolers are an age group highly vulnerable to the risks of the digital environment. A significant number of parents try to establish family rules for the use of digital devices, but most children either cannot identify these rules, or do not know about their existence at all. Additionally, many parents of preschoolers do not know what strategies and means should be applied in situations where their child faces online risks.

## Conclusion

Taking into account the current data on the use of digital devices by children (increasing duration, scope, and purpose of use), it is necessary to talk about establishing the foundations of digital competence of preschool children, and determining its content in accordance with the children’s age characteristics.

Our study made an attempt to describe the content of the components of digital competence in older preschool children. We found that, despite the fact that the children in our study had basic skills in using digital devices such as smartphones and tablets, the majority had little or no knowledge of the application of these technologies in the life of society, and their impact on the environment and the community as a whole. The children successfully used digital technologies for entertainment purposes, which, together with limited knowledge, negatively affected their motivation to develop the skills to use them in other areas. Mastery of the digital world was gained by these older preschoolers mainly spontaneously, without proper support from adults; thus, the children were deprived of the opportunity to get the necessary ideas about safe behavior in the digital environment. Preschoolers actively explored the entertainment possibilities of digital devices, often going beyond child-appropriate content and age-appropriate environments. In the process, they often faced various online risks. However, due to the existing problems in mediating the children’s use of digital devices, their parents may not have any idea of the difficulties or negative emotions their child experiences in the digital environment.

As we continue this study, we plan to increase the sample with representatives from different regions of the country. This will take into account the different ethnic, socio-economic, and other characteristics of the population. It is also necessary to compare the features of digital competence of preschoolers relative to the educational and economic status, and composition of the family — in particular, the presence of siblings. In our study, several respondents mentioned their siblings’ role in familiarizing them with digital devices and moderating their use; thus, having siblings may be an important factor in the development of digital competence, but this study was not able to take this into account.

## Limitations

This study had some limitations. Generalization of the results is limited by the small sample size. Socio-stratification characteristics of the families were not taken into account. Mainly mothers of children participated in the survey. Interviewing both parents, whenever possible, can provide significantly more information about the digital environment and competence of a child.

## References

[ref1] Archer, K., Wood, E., & De Pasquale, D. (2021). Examining joint parent-child interactions involving infants and toddlers when introducing mobile technology. Infant Behavior and Development, 63, 101568. 10.1016/j.infbeh.2021.101568 33906028

[ref2] Batenova, Iu.V., Emel’ianova, I.E., Ivanova, I.Iu., Filippova, O.G., & Chumicheva, R.M. (2019). Informatsionnaia gramotnost’ detei doshkol’nogo vozrasta: sushchnost’, spetsifika, opyt [Information literacy of preschool children: Essence, specificity, experience]. Cheliabinsk: Izdatel’skii tsentr “Titul”.

[ref3] Bochkarev, A.V. (2019). Vliianie tekhnologii virtual’noi real’nosti na razvitie tvorcheskogo potentsiala odarennogo rebenka [Influence of virtual reality technologies on the development of creative potential of a gifted child]. Nauchnyi Al’manakh [Science Almanac], 12–2(62), 28–31. Retrieved from https://ukonf.com/doc/na.2019.12.02.pdf

[ref4] Brito, R., & Dias, P. (2019). Technologies and children up to 8 years old: what changes in one year? Observatorio, 13(2), 68–86. 10.15847/obsOBS13220191366

[ref5] Calvani, A., Cartelli, A., Fini, A., & Ranieri, M. (2008). Models and instruments for assessing digital competence at school. Journal of E-learning and Knowledge Society, 4(3), 183–193. 10.20368/1971-8829/288

[ref6] Carretero, G.S., Vuorikari, R., & Punie, Y. (2017). DigComp 2.1: The Digital Competence Framework for Citizens with eight proficiency levels and examples of use. Publications Office of the European Union. https://publications.jrc.ec.europa.eu/repository/handle/JRC101254

[ref7] Chaudron, S., Di Giota, R., & Gemo, M. (2018). Young Children (0–8) and Digital Technology, a Qualitative Study across Europe. Publications Office of the European Union. https://publications.jrc.ec.europa.eu/repository/handle/JRC110359

[ref8] Common Sense Media. (2013). Zero to eight: Children’s media use in America. https://www.commonsensemedia.org/research/past-research-reports

[ref9] Cortesi, S., Hasse, A., Lombana-Bermudez, A., Kim, S., & Gasser, U. (2020). Youth and Digital Citizenship+ (Plus): Understanding Skills for a Digital World. Berkman Klein Center for Internet & Society. 10.2139/ssrn.3557518

[ref10] Dardanou, M., Unstad, T., Brito, R., Dias, P., Fotakopoulou, O., Sakata, Y., & O’Connor, J. (2020). Use of touchscreen technology by 0–3-year-old children: Parents’ practices and perspectives in Norway, Portugal and Japan. Journal of Early Childhood Literacy, 20(3), 551–573. 10.1177/1468798420938445

[ref11] Denisov, D.V. (2018, April). Ot tsifrovoi gramotnosti k tsifrovoi kompetentnosti [From digital literacy to digital competence]. Paper presented at the International Scientific and Practical Conference Pedagogical and Sociological Aspects of Education, Cheboksary. Retrieved from https://phsreda.com/ru/article/11033/discussion_platform

[ref12] Dore, R.A., & Dynia, J.M. (2020). Technology and Media Use in Preschool Classrooms: Prevalence, Purposes, and Contexts, Frontiers in Education (Frontiers), 5, 244. https://doi.org/0.3389/feduc.2020.600305

[ref13] El’koninova, L.I., & Grigor’ev, I.S. (2015). Opyt i perspektivy issledovaniia detskoi igry v ramkakh kul’turno-istoricheskoi psikhologii [Experience and perspectives of the study of children’s play in the framework of cultural and historical psychology]. Kul’turno-istoricheskaia psikhologiia [Cultural-Historical Psychology], 11(3), 16–24. 10.17759/chp.2015110303

[ref14] Ferrari, A. (2013). DIGCOMP: A Framework for Developing and Understanding Digital Competence in Europe. Publications Office of the European Union. https://publications.jrc.ec.europa.eu/repository/handle/JRC83167

[ref15] Hoffman, B. (2014). Computer as a Threat or an Opportunity for Development of Children. Procedia — Social and Behavioral Sciences, 146, 15–21. 10.1016/j.sbspro.2014.08.080

[ref16] Ilomäki, L., Paavola, S., Lakkala, M., & Kantosalo, A. (2016). Digital competence — an emergent boundary concept for policy and educational research. Education and Information Technologies, 21(3), 655–679. 10.1007/s10639-014-9346-4

[ref17] Karabanova, O.A., & Molchanov, S.V. (2018). Riski negativnogo vozdeistviia informatsionnoi produktsii na psikhicheskoe razvitie i povedenie detei i podrostkov [Risks of the negative impact of information products on the mental development and behavior of children and adolescents]. Natsional’nyi psikhologicheskii zhurnal [National Psychological Journal], 3(31), 37–46. 10.11621/npj.2018.0304

[ref18] Khanova, T.G., & Semenova, E.V. (2019). Problema «vzaimodeistviia» detei doshkol’nogo vozrasta s mobil’nymi ustroistvami [The problem of “interaction” of preschool children with mobile devices]. Gosudarstvennyi sovetnik [Economic Consultant], 1, 85–90. Retrieved from https://gossovetnik.files.wordpress.com/2019/03/190114.pdf

[ref19] Kim, Y., & Smith, D. (2017). Pedagogical and technological augmentation of mobile learning for young children’s interactive learning environments. Interactive Learning Environments, 25, 4–16. 10.1080/10494820.2015.1087411

[ref20] Kluzer, S., & Pujol, P.L. (2018). DigComp into Action: Get inspired, make it happen. A user guide to the European Digital Competence Framework. Publications Office of the European Union. https://publications.jrc.ec.europa.eu/repository/handle/JRC110624

[ref21] Korotkova, A., Verlina, Iu., Tsai, T., Nikitina, S., Sosnovaia, A., & Khokhlova, A. (2018). Deti. Mediapotreblenie. 2017 [Children. Media consumption. 2017]. Moscow: MOMRI — Modern Media Research Institute.

[ref22] Liu, W., Tan, L., Huang, D., Chen, N., & Liu, F. (2021). When preschoolers use tablets: The effect of serious educational games on children’s attention development. International Journal of Human–Computer Interaction, 37(3), 234–248. 10.1080/10447318.2020.1818999

[ref23] Papadakis, S. (2021). Tools for evaluating educational apps for young children: A systematic review of the literature. Interactive Technology and Smart Education, 18(1), 18–49. 10.1108/ITSE-08-2020-0127

[ref24] Papadakis, S., Alexandraki, F. & Zaranis, N. (2021). Mobile device use among preschool-aged children in Greece. Educ Inf Technol, 6, 1–34. Retrieved from https://link.springer.com/article/10.1007/s10639-021-10718-6 10.1007/s10639-021-10718-6 PMC8406380 34483704

[ref25] Przybylski, A.K., & Weinstein, N. (2017). Digital Screen Time Limits and Young Children’s Psychological Well-Being: Evidence from a Population-Based Study. Child Development, 90 (1), 56–65. 10.1111/cdev.13007 29235663

[ref26] Radesky, J.S., Schumacher, J., & Zuckerman, B. (2015). Mobile and interactive media use by young children: The good, the bad, and the unknown. Pediatrics, 135(1), 1–3. 10.1542/peds.2014-2251 25548323

[ref27] Radesky, J.S., & Christakis, D.A. (2016). Increased Screen Time: Implications for Early Childhood Development and Behavior. Pediatric Clinics of North America, 63(5), 827–839. 10.1016/j.pcl.2016.06.006 27565361

[ref28] Recommendation of the European Parliament and of the Council of 18 December 2006 on key competences for lifelong learning. Official Journal of the European Union. Retrieved from http://data.europa.eu/eli/reco/2006/962/oj

[ref29] Soldatova, G.U., & Vishneva, A.E. (2019). Features of the Development of the Cognitive Sphere in Children with Different Online Activities: Is There a Golden Mean? Konsul’tativnaya psikhologiya i psikhoterapiya [Counseling Psychology and Psychotherapy], 27(3), 97–118. 10.17759/cpp.2019270307

[ref30] Soldatova, G.U., Nestik, T.A., Rasskazova, E.I., & Zotova, E.Iu. (2013). Tsifrovaia kompetentnost’ rossiiskikh podrostkov i roditelei: Rezul’taty vserossiiskogo issledovaniia [Digital competence of Russian teenagers and parents: Results of the all-Russian study]. Moscow: Fond Razvitiia Internet.

[ref31] Tisseron, S. (2013). Les dangers de la télé pour les bébés. Bruxelles: Yapaka.be.

[ref32] UNICEF (2017). The State of the World’s Children 2017: Children in a Digital World. https://www.unicef.org/media/48601/file

[ref33] United Nations Committee on the Rights of the Child (2021). Convention on the Rights of the Child. General comment No. 25 (2021) on children’s rights in relation to the digital environment. https://tbinternet.ohchr.org/_layouts/15/treatybodyexternal/Download.aspx?symbolno=CRC/C/GC/25&Langen

[ref34] Veraksa, A.N., & Bukhalenkova, D.A. (2017). Primenenie komp’iuternykh igrovykh tekhnologii dlia razvitiia reguliatornykh funktsii doshkol’nikov [Computer game-based technology in the development of preschoolers’ executive functions]. Rossiiskii Psikhologicheskii Zhurnal [Russian Psychological Journal], 14(3), 106–133. 10.21702/rpj.2017.3.6

[ref35] Veraksa, A.N., Bukhalenkova, D.A., Chichinina, E.A., & Almazova, O.V. (2020). Osobennosti ispol’zovaniia tsifrovykh ustroistv sovremennymi doshkol’nikami [Digital Devices Use by 6–7 Years-Old Children]. Sotsiologicheskie Issledovaniia [Sociological Research], 6, 82–92. 10.31857/S013216250009455-3

[ref36] Vuorikari, R., Punie, Y., Carretero, G.S., & Van den Brande, G. (2016). DigComp 2.0: The Digital Competence Framework for Citizens. Update Phase 1: The Conceptual Reference Model. Publications Office of the European Union. https://publications.jrc.ec.europa.eu/repository/handle/JRC101254

